# Role of immunotherapy in treatment refractory chordomas: review of current evidence

**DOI:** 10.3389/fsurg.2024.1375567

**Published:** 2024-05-30

**Authors:** A. Yohan Alexander, Sanjay Dhawan, Andrew S. Venteicher

**Affiliations:** Department of Neurosurgery and Center for Skull Base and Pituitary Surgery, University of Minnesota, Minneapolis, MN, United States

**Keywords:** chordoma, metastasis, spine, skull base, immunotherapy, oncology, tumor

## Abstract

**Introduction:**

Chordomas are aggressive tumors that are thought to arise from remnants of the embryological notochord. They can arise along the ventromedial aspect of the sacrum, mobile spine, and clivus—with most cases occurring in the sacrum or skull base. Despite surgery and radiation, chordomas often progress and become refractory to further treatment. The high recurrence rate of chordomas has created an urgent need to develop new systemic treatment options. Recent case reports and clinical trials have highlighted the use of immunotherapy for refractory chordomas. In this review, we summarize the results of these studies and discuss the potential role of immunotherapy for chordomas.

**Methods:**

The PUBMED database was queried for studies mentioning both “Chordoma” and “Immunotherapy.” All case series and case reports that involved administration of an immunotherapy for chordoma were included. Additional studies that were found during literature review were added. ClinicalTrials.Gov was queried for studies mentioning both “Chordoma” and “Immunotherapy.” The final cohort consisted of all clinical trials that utilized immunotherapy for chordomas of any location.

**Results:**

Eight case reports and series detailing the use of immunotherapy for treatment refractory chordoma were identified. Most patients received immunotherapy targeting the PD-1/PD-L1 interaction, and two patients received therapy targeting this interaction along with the tyrosine kinase inhibitor pazopanib. One patient received a vaccine derived from autologous tumor cells, and one patient received a viral vector that downregulated the effect of TGF-beta. One clinical trial utilized a brachyury vaccine in conjunction with standard of care radiotherapy.

**Conclusions:**

Immunotherapy for chordoma is a promising area of investigation with increasing, but small, numbers of case series and clinical trials. Despite challenges in patient accrual, future directions in chordoma immunotherapy may lie in vaccine-based therapies and immune checkpoint inhibitors. Understanding chordoma heterogeneity and microenvironment will likely elucidate important chordoma features that will inform future clinical trial design.

## Introduction

Chordomas are aggressive tumors that are thought to arise from remnants of the embryological notochord. They can arise along the ventromedial aspect of the sacrum, mobile spine, and clivus—with most cases occurring in the sacrum or skull base (1–3). Chordomas are divided into three histological subtypes including conventional, dedifferentiated, and poorly differentiated (4). The initial treatment of choice for chordoma is maximal safe resection, and radiation therapy in select cases. While gross total resection significantly prolongs the progression free and overall survival for patients with chordoma, extent of resection can be limited in cases where critical neurovascular structures are involved (5–7). In the setting of subtotal resection, focused radiation may be useful, but the high doses of radiation required raises the risk of radiation-induced injury to the brainstem and spinal cord (8). Despite surgery and radiation, many chordomas progress to become unresectable, or may be too diffuse for radiation.

While there is an urgent need for systemic therapy for patients with recalcitrant chordoma, efficacy of traditional chemotherapy against chordomas has not proven useful, and targeted molecular therapies thus far offer limited benefit (9). Few recent case reports and clinical trials have highlighted the use of immunotherapy for treatment of refractory chordomas. Though no strict guidelines exist, indications for the utilization of immunotherapy for chordoma have been limited to lesions that have been previously treated with surgery and radiation but have progressed to become radioresistant and unresectable. In this review, we summarize the results of these exploratory studies and discuss the potential future role of immunotherapy for chordomas.

## Materials and methods

### Case series and case reports

**Figure 1:**
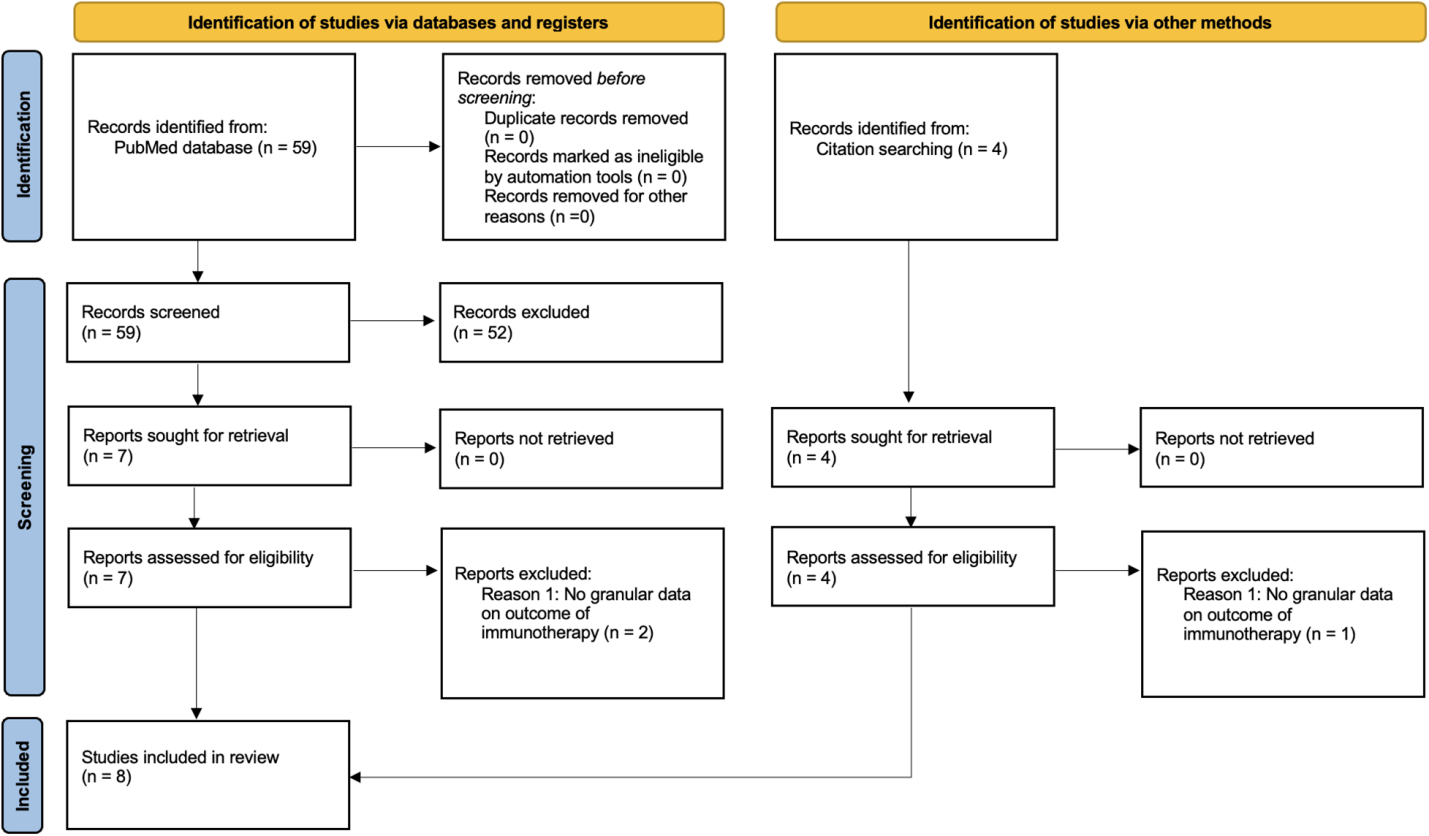
PRISMA flow diagram detailing selection of case reports and case series.

The PUBMED database was queried for studies mentioning both “Chordoma” and “Immunotherapy” per PRISMA guidelines. Studies that utilized immunotherapy for the treatment of recalcitrant chordoma and reported outcomes regarding tumor control rates or overall survival were included. As most studies were case reports and case series, a comparison group was not required for inclusion. All case series and case reports that involved administration of an immunotherapy for chordoma were included. Studies that did not provide the outcome of immunotherapy were excluded. Additional studies that were identified during literature review but were not results of the PUBMED search were also added. Variables collected included age at initial chordoma diagnosis, sex, initial chordoma location, disease stage (classified as either localized, locally progressive, or metastatic), time from initial diagnosis to immunotherapy administration, type of immunotherapy, and the clinical and radiographic outcome of the immunotherapy. A flow chart detailing selection of case reports and case series can be seen in Figure 1.

### Clinical trials

**Figure 2:**
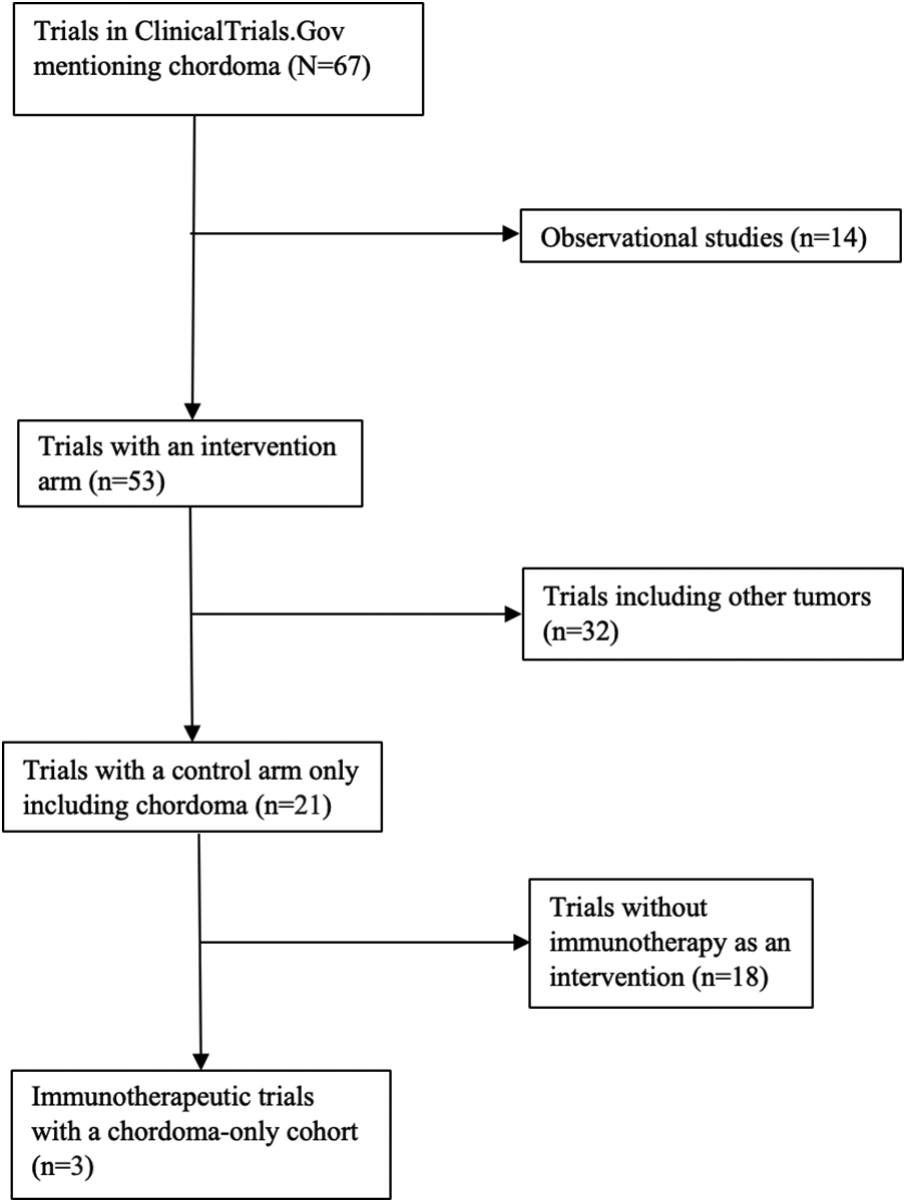
Selection of clinical trials utilizing immunotherapy for chordoma.

ClinicalTrials.Gov was queried for studies mentioning “Chordoma.” Observational studies, studies that included other solid tumors and sarcomas, and studies without any planned enrollment were excluded. The final cohort consisted of all clinical trials that utilized immunotherapy for chordomas of any location. Cohort and intervention details of interest included estimated enrollment size, disease stage (classified as locally progressive or metastatic), location of initial chordoma, type of immunotherapy utilized, and, if applicable, clinical and radiographic outcome. A flow chart detailing selection of clinical trials can be seen in Figure 2.

## Results

### Case series and case reports

An early case series demonstrated the potential role of various immunotherapeutic strategies for three recurrent and heavily pretreated chordomas (10). All patients had several prior surgeries and radiation. One patient was treated with cetuximab eight years after diagnosis, which resulted in 4 months of progression-free survival (PFS). Eleven years after diagnosis, the patient was treated with pembrolizumab which resulted in a significant decrease in size of the chordoma, improvement of the patient's facial palsy secondary to decreased mass effect, and no progressive disease at 6-month follow-up. Another patient was treated with a vaccine derived from autologous, irradiated tumor cells as well as capsules releasing granulocyte-macrophage colony-stimulating factor (GM-CSF) every seven days. The patient had no progression of chordoma over the 19-month period of follow-up. Of note, this treatment was part of a clinical trial looking at the efficacy of MVX-ONCO-1 on solid tumors (ClinicalTrials.gov identifier: NCT02193503). A third patient was treated with nivolumab after several surgeries, proton beam therapy, and pazopanib. A clinical and radiographic response were observed for 6 months, but the patient had progressive disease at 6-month follow-up.

A second case series focused on PD-1 (programmed cell death protein 1)/PD-L1 (programmed cell death protein ligand 1) inhibition in recurrent chordoma (11). Agents in this study include pembrolizumab, durvalumab, nivolumab, and FAZ053, all of which target the PD-1/PD-L1 interaction. One patient had a complete response, three patients had a partial response, 11 patients had stable disease, and two patients had progressive disease. The median PFS was 14 months, and, at last follow-up, one patient had no residual chordoma, 12 patients were alive with residual chordoma, and four patients had died.

Four additional case reports have described the use of pembrolizumab or nivolumab, both of which inhibit the PD-1/PD-L1 interaction, for recurrent chordoma (12–15). One patient with recurrent metastatic chordoma of the sacrum was placed on pembrolizumab and had over a 30% reduction in the number of metastatic nodules and tumor burden. Nine months after initiation of pembrolizumab, their chordoma progressed, leading to discontinuation of immunotherapy (12). A second patient with Lynch syndrome and recurrent clival chordoma received pembrolizumab and had a radiographic and clinical response, reflecting the potential link between high total mutational burden and immunotherapy efficacy (13). A third patient received pembrolizumab after two surgeries, proton beam radiotherapy, and stereotactic radiosurgery, but discontinued after 12 weeks due to immune-related adverse. After further failed therapy with imatinib and abemaciclib, the patient was treated with everolimus, which controlled his disease for 6-months with no sign of progression (14). A fourth patient with an INI1-deficient clival chordoma received nivolumab after receiving 4 cycles of vincristine, doxorubicin and cyclophosphamide with alternating ifosfamide and etoposide (15). After tumor progression following chemotherapy, the patient received nivolumab and had marked 58% reduction of tumor volume. At the time of the 14th cycle of nivolumab, the patient's tumor size returned to the pre-nivolumab baseline, but the patient continued with nivolumab as it provided clinical benefit with regard to functional independence and quality of life.

One case series of clival chordomas treated at a single institution detailed a patient who received a combination of nivolumab, and pazopanib, a tyrosine kinase inhibitor with broad activity (16). After three prior surgeries and proton beam radiotherapy, the patient was placed on the combination of nivolumab and pembrolizumab which resulted in PFS for the 14 months of reported follow-up.

Alternative strategies towards immunotherapeutic treatment of chordoma have also been reported. A patient with metastatic sacral chordoma previously failing chemotherapy and nivolumab was trialed on AdAPT-001, an oncolytic virus delivery of a TGF-beta trap to neutralize the immunosuppressive cytokine TGF-beta (17). The chordoma that had metastasized to the lung was injected directly with AdAPT-001 and the lesion remained stable. The patient's restrictive pulmonary symptoms had improved. The patient then was treated with pembrolizumab which resulted in reduction in radiographic tumor burden for seven months, at which point the lesion progressed.

The details of case series and case reports utilizing immunotherapy for chordoma can be found in Table 1.

**Table 1:** Overview of case reports and series using immunotherapy for chordoma.

Study	# of patients	Age at diagnosis	Sex	Initial location	Disease stage	Immunotherapy (Years from diagnosis)	Target	Outcome
Migliorini et al. (10)	3	Patient 1:67 Patient 2: 49 Patient 3: 40	Patient 1: F Patient 2: M Patient 3: F	Patient 1: Cervical Patient 2: Clival Patient 3: Clival	Patient 1: Metastatic Patient 2: Locally Progressive Patient 3: Locally progressive	Patient 1: Cetuximab (8) followed by pembrolizumab (11) Patient 2: Vaccine with irradiated autologous tumor cells (4) Patient 3: nivolumab (16)	Patient 1: EGFR and PD-1, respectively Patient 2: Tumor Patient 3: PD-1	Patient 1: 4 months PFS, 6 months PFS and reduction in tumor size Patient 2: 19 months PFS and reduction in tumor size Patient 3: 9 months PFS and reduction in tumor size
Wu et al. (12)	1	52	M	Sacral	Metastatic	Pembrolizumab (1)	PD-1	9 months PFS and reduction in metastasis size at 4 months
Bishop et al. (11)	17	Median: 56	M: 13 F: 4	Sacral: 10 Mobile spine: 5 Clival: 2	Localized: 5 Locally progressive: 5 Metastatic: 7	Pembrolizumab (NA): 9 patients Durvalumab (NA): 5 patients FAZ053 (NA): 2 patients Nivolumab (NA): 1 patient	PD-1 PD-1 PD-L1 PD-1	Stable disease: 11 Partial response: 3 Complete response: 1 Progressive disease: 2 Median PFS: 14 months
Shinojima et al. (13)	1	72	M	Clival	Progressive and nonmetastatic	Pembrolizumab (2)	PD-1	Reduction in tumor size
Kesari et al. (17)	1	66	NA	Sacral	Metastatic	Oncolytic virus followed by pembrolizumab (NA)	PD-1	7 months PFS and reduction in tumor size
Ibodeng et al. (14)	1	77	M	Clival	Progressive and nonmetastatic	Pembrolizumab (9)	PD-1	Discontinued at 12 weeks secondary to immune related adverse events
Jägersberg et al. (16)	1	31	F	Clival	NA	Nivolumab with pazopanib (NA)	PD-1	14 months PFS
Williamson et al. (15)	1	NA	NA	Clival	Metastatic	Nivolumab (NA)	PD-1	58% tumor volume reduction at three cycles with regrowth to baseline by cycle 14. Continued clinical benefit at last follow-up

EGFR, epidermal growth factor receptor; F, female; M, male; NA, not available; PD-1, programmed cell death protein; PD-L1, Programmed cell death protein ligand 1; PFS, progression-free survival.

### Clinical trials

Clinical trials to determine efficacy of immunotherapy-based strategies in the treatment of chordoma are a high priority. Three clinical trials meeting our predefined search criteria were identified. Of these, only one had published results. An exciting therapeutic avenue in the treatment of chordoma has centered around the idea that the brachyury transcription factor is expressed in chordoma but not in virtually all other normal tissues. Therefore, brachyury represents a tumor-specific target attractive for vaccine-based therapies. A yeast brachyury vaccine (GI-6301) in combination with radiation was trialed for locally advanced, unresectable, and non-metastatic, chordoma (18). Of 24 patients included, 11 patients received the vaccine and 13 patients received placebo, with all patients receiving radiotherapy. Both arms had mounted similar T-cell immune responses to brachyury, and no differences in radiographic or clinical response were seen. Given the low efficacy of the vaccine compared to the control arm, the trial was discontinued.

A summary of clinical trials utilizing immunotherapy for chordoma can be found in Table 2.

**Table 2:** Overview of clinical trials using immunotherapy for unresectable chordoma.

Study name	Trial number	Sample size	Disease stage	Location	Immunotherapy	Target	Outcome
Nivolumab and Relatlimab in Treating Participants With Advanced Chordoma	NCT03623854	10	Metastatic	All locations	Nivolumab and relatimab	PD-1 and LAG-3, respectively	Unpublished
Nivolumab With or Without Stereotactic Radiosurgery in Treating Patients With Recurrent, Advanced, or Metastatic Chordoma	NCT02989636	21	Progressive or metastatic	All locations	Nivolumab	PD-1	Unpublished
QUILT-3.011 Phase 2 Yeast-Brachyury Vaccine Chordoma (18)	NCT02383498	24	Progressive and non-metastatic	All locations	Brachyury vaccine	Brachyury	Vaccine arm: 5 progressive disease, 3 stable disease, 1 partial response, 2 non-evaluable Placebo arm: 8 progressive disease, 4 stable disease, 1 partial response

EGFR, epidermal growth factor receptor; LAG-3, Lymphocyte activation gene-3; PD-1, programmed cell death protein.

## Discussion

**Figure 3:**
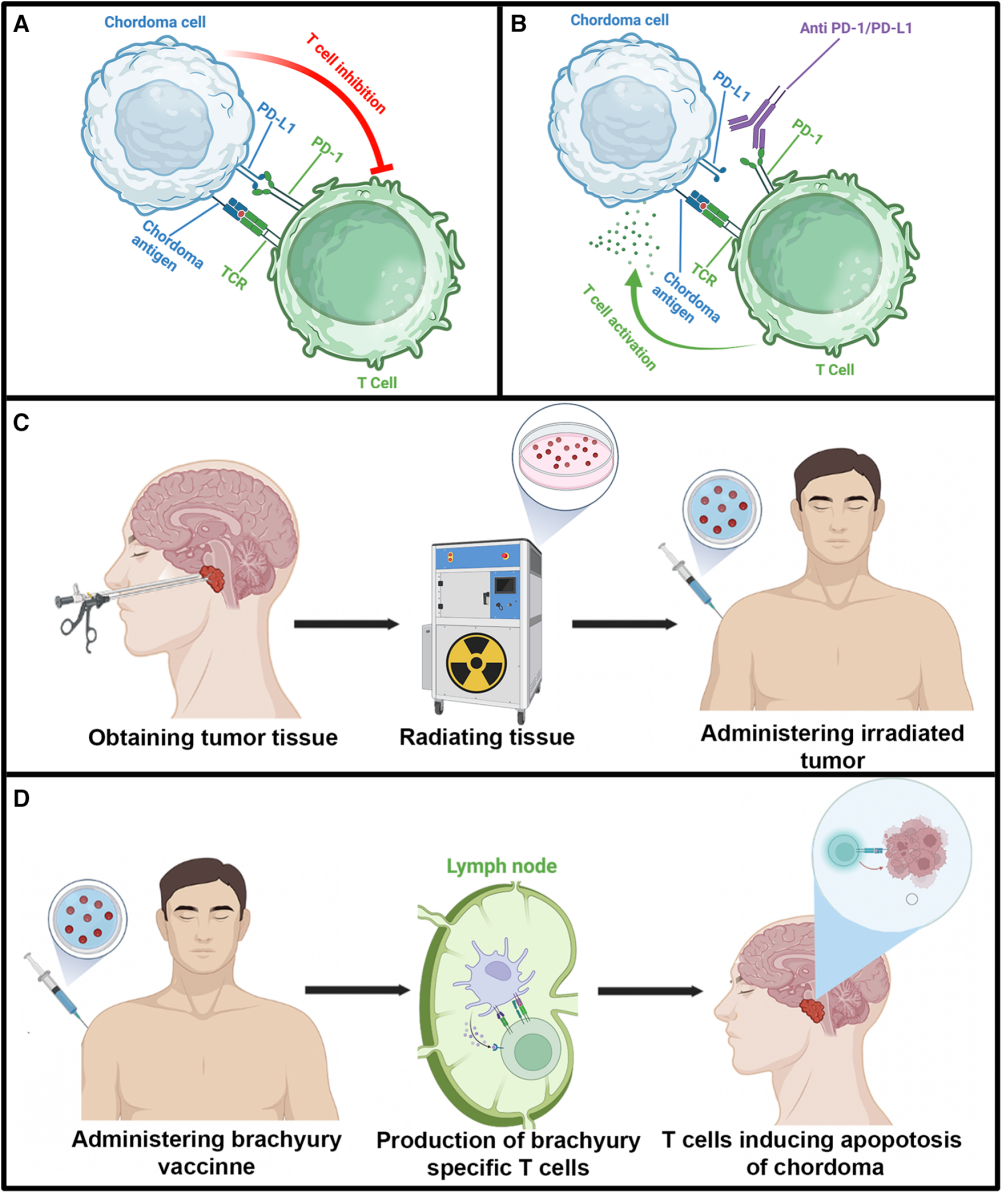
Illustration depicting immunosuppressive activity of the PD/PD-L1 interaction and various immunotherapeutic strategies reported in the literature for chordoma. Illustrations in each panel were created with Biorender.com. (**A**) T cell inhibition caused by the PD/PD-L1 interaction. (**B**) Inhibition of PD/PD-L1 immunosuppression by pembrolizumab. (**C**) Diagram detailing the utilization of autologous irradiated chordoma. (**D**) Diagram demonstrating brachyury vaccine. TCR, T cell receptor.

The utilization of immunotherapy for chordoma is a critical area of future research, and so far, several case series and clinical trials have been reported using a variety of immunotherapy strategies (Figure 3). Among case reports and series, most patients received pembrolizumab or a similar immunotherapeutic agent targeting the PD-1/PD-L1 interaction. For most patients, this targeted immunotherapy resulted in tumor regression and symptom improvement for a few months until the tumor eventually progressed, and immunotherapy was halted. Methods of creating more sustainable responses with immunotherapy will be a necessary topic of future investigation. In rare patients, sustainable responses were seen, as one patient in a sarcoma clinical trial received vaccine of irradiated, autologous tumor and was recurrence free at 19-month follow-up (10). While limited recommendations can be suggested due to the literature being largely restricted to case reports and small case series, immunotherapy is being considered for progressive chordomas that are unresectable and have failed radiation in the past. When immunotherapy is employed, the PD-1/PD-L1 interaction has been utilized the most and may provide tumor control in a subset of patients. Further, adverse effects due to the immunotherapies reported in the literature are not common.

Despite the application of immunotherapy to many types of cancers, the relationship between immunotherapy response and tumor microenvironment is still being defined. Studies elucidating the immune microenvironment of chordomas have shown that PD-L1 is present in tumor associated lymphocytes, but not in chordoma tumor cells (19). Other studies have shown that chordomas that expressed galectin-9 were also associated with more locally aggressive behavior, which correlated with a lower functional status for the patients (20). The expression of both galectin-9 and PD-L1 are pro-apoptotic for tumor invading lymphocytes and may allow for the chordoma to escape the patient's anti-tumor immune response. Apart from tumor infiltrating lymphocytes expressing PD-L1/PD-1 and chordoma cells producing galectin-9, many chordomas may not be strongly immunogenic (20). For example, despite success with brachyury silencing in-vitro in preclinical models, in the clinical trial using a brachyury vaccine plus radiation, the vaccine arm had no benefit over the placebo arm regarding tumor progression and the trial was discontinued (18, 21, 22). Though early, the sarcoma clinical trial utilizing autologous tumor as a vaccine source led to PFS for 19 months in one chordoma patient (10). These studies have highlighted the benefit of studying the immune microenvironment of chordoma, and recent single cell genomics and spatial transcriptomics studies have revealed new understanding on how the immune system can be modulated in chordoma (23–25).

PD-1/PD-L1 targeted therapies holds promise as these proteins are often expressed in tumor associated lymphocytes (19, 20). For future directions with vaccines, apart from brachyury and autologous tumor vaccines, other proteins expressed in chordoma that have been thought to represent useful targets include S100 and epithelial membrane antigen. High molecular weight-melanoma associated antigen may be beneficial to target as its expression within the tumor has been to significantly increase the risk of death and metastasis in patients with chordomas (26, 27). Since chordomas are largely thought to not be highly immunogenic due to an absence of mutational burden, vaccine based therapies may be needed in addition to immune checkpoint inhibitors (28, 29). Despite promising targets and future steps regarding further exploration of immunotherapy in chordoma, the rare incidence of this entity makes it challenging to study for clinical trials where large numbers of patients are necessary. The molecular heterogeneity of chordoma and lack of widely accepted biomarkers to create molecularly informed clinical trials is also a limitation with current trial design in chordoma (30–33). Due to the challenges in studying the therapeutic efficacy in chordoma patients, there is a great emphasis on the development of preclinical models for laboratory-based testing. More cell lines and xenografts are being created among the chordoma research community, which are particularly important given the current lack of a chordoma mouse model.

Alternative methods for treating unresectable, radioresistant chordomas include targeted molecular therapy. Commonly reported molecular targets in chordoma include platelet derived growth factor (PDGFR), vascular endothelial growth factor receptor (VEGFR), and epidermal growth factor receptor (EGFR), insulin-like growth factors (IGF), and mammalian target of rapamycin (mTOR). PDGFR targeting agents include imatinib and dasatinib. In a phase II clinical trial in recurrent PDGFR positive chordomas, imatinib treatment resulted in stable disease for 70% of patients and the median PFS was nine months (34). Imatinib used in patients with recurrent PDGFR positive chordomas resulted in stable disease for 74% of patients and the median PFS was nine months (35). No patients had partial or total reduction in their tumor burden. Other studies have looked at molecular inhibitors that target PDGFR along with other receptors. One basket study looking at the efficacy of dasitinib; an inhibitor of PDGFR c-KIT, BCR-ABL, and ephrin receptor kinases; in chordoma found that the 6-month, 2-year, and 5-year overall survival for patients was 54%, 43%, and 18% respectively (36). In a series of four patients treated with pazopanib; which targets PDGFR, VEGFR, c-kit, and fibroblast growth factor receptor (FGFR); and sunitinib, which targets PDGFR and VEGFR, four patients were treated with pazopanib and had a mean PFS of 8.5 months. The one patient treated with sunitinib had radiographic regression of her metastatic chordoma and had disease stabilization for 27 months (37). EGFR targeted therapies utilized in advanced chordoma include lapitinib and erlotinib. In a phase II study, 33% of patients had partial response and 39% of patients had stable disease (38). Additionally, erlotinib has proven to be effective against chordoma that was previously recalcitrant to imatinib (39, 40). One study reports the use of lisitinib, an IGF-1 receptor targeting agent, in a patient with chordoma which resulted in stable disease for five years (41). Everolimus and imatinib have been used for patients who had chordomas resistant to imatinib monotherapy (42). At 12-months, 48.1% of patients experienced continued control of their chordoma. Of note, approximately 30% of patients discontinued therapy due to adverse events. Patients with chordoma resistant to imatinib monotherapy had favorable response to imatinib combined with sirolimus as 89% of patients had clinical benefit as defined by the RECIST criteria (43). While VEGFR targeting therapies including sorafenib and thalidomide have also been utilized, a high rate of adverse reactions precludes its frequent use in patients (37, 44). Conventional chemotherapies have not proven useful for chordoma (9).

## Conclusion

Immunotherapy for chordoma is a promising area of investigation with increasing, but small, numbers of case series and clinical trials. Despite challenges in patient accrual, future directions in chordoma immunotherapy may lie in vaccine-based therapies and immune checkpoint inhibitors. Understanding chordoma heterogeneity and microenvironment will likely elucidate important chordoma features that will inform future clinical trial design.
